# Investigation of ejaculatory disorder by silodosin in the treatment of prostatic hyperplasia

**DOI:** 10.1186/1471-2490-12-29

**Published:** 2012-10-19

**Authors:** Koichi Sakata, Tatsuo Morita

**Affiliations:** 1 Department of Urology, Imaichi Hospital, 381 Imaichi, Tochigi, Nikko-shi, Japan; 2 Department of Urology, Faculty of Reno-urology Surgery, Jichi Medical University, 3311-1 Yakushiji, Tochigi, Shimotsuke-shi, Japan

**Keywords:** Silodosin, α1 blocker, Ejaculatory disorder, Adverse reaction, Sexual action

## Abstract

**Background:**

To assess the ejaculatory disorder caused by silodosin in the prostatic hyperplasia patients who carry out sexual actions (sexual intercourse, masturbation).

**Method:**

The subjects of this study were 91 patients who had been clinically diagnosed to have LUTS/BPH at this hospital, who were administered silodosin at 4 mg twice a day, and who gave response to a questionnaire survey related to ejaculatory disorder. Sexual intercourse and masturbation were regarded as sexual actions in this study.

**Results:**

Ejaculatory disorder occurred in 38 (42%) of the 91 silodosin administration cases. Forty (44%) of the 91 patients answered that they carried out sexual actions after oral intake of silodosin. When the investigation was conducted only in those who exercised sexual actions, ejaculatory disorder was observed in 38 (95%) of these 40 patients, indicating a high incidence. When asked if disturbed by the ejaculatory disorder, 29 (76%) of the 38 patients who had ejaculatory disorder answered yes. Oral silodosin was discontinued due to the ejaculatory disorder in 2 (5%) of these patients. On the whole, the discontinuation rate of oral silodosin was 2% (2/91 patients).

**Conclusion:**

It was demonstrated that the administration of silodosin induced ejaculatory disorder at a high incidence. Since it is possible that the high frequency of ejaculatory disorder by silodosin may reduce QOL, it is considered necessary to provide sufficient information related to ejaculatory disorder at the time of treatment with silodosin.

## Background

Sympathetic nerve α1 receptor has receptor subtypes α1A, α1B and α1D. Recently, the research on α1A receptor subtype has made much progress so that various drugs to treat LUTS by BPH have been commercialized [1,2]. The affinity of tamuslosin hydrochloride to α1A receptor is comparatively high while naftopidil is characterized to have comparatively high affinity to α1D receptor subtype [3,4]. On the other hand, silodosin acts on α1A receptor in a very specific manner [5]. The practical clinical application of silodosin started in Japan in 2006. However, this drug frequently causes ejaculatory disorder as an adverse reaction. The incidence of ejaculatory disorder in the phase III clinical study in Japan is reported as 22.3% [6]. However, this incidence of ejaculatory disorder refers to the incidence in the patients treated with silodosin on the whole without taking the presence or absence of sexual actions (sexual intercourse, masturbation) into consideration. The true incidence of ejaculatory disorder should be calculated by investigating the patients who carried out sexual actions during the administration of α1 blocker. Accordingly, a questionnaire survey related to the ejaculatory disorder was conducted this time in the LUTS/BPH patients under treatment with silodosin to investigate the circumstance of ejaculatory disorder caused by silodosin among the patients who exercised sexual actions.

## Methods

The subjects of this study were 91 patients who had been clinically diagnosed to have LUTS/BPH at this hospital between June 2006 and July 2011, who were administered silodosin at 4 mg twice a day, and who gave response to a questionnaire survey retrospectively (Table 1). In this regard, the standard oral dose of silodosin is 8 mg/day in Japan. Sexual intercourse and masturbation were regarded as sexual actions in this study.

**Table 1:** Questionnaire survey table

Question 1:
Have you experienced the following symptoms since you started taking the drug?
* Loose stools and diarrhea.
* Dizziness.
* Light-headed feeling when standing up or changing the posture.
* Headache.
* Nasal congestion.
* Feel thirst.
* Decreased amount of semen or a feeling different from that in the past at the time of ejaculation.
Question 2:
How often do you carry out sexual actions (sexual intercourse, masturbation)?
* Not at all.
* About ( ) times in (1 month, 3 months, 6 months, 1 year).
The following questions are only for those who have carried out sexual actions (sexual intercourse, masturbation) since the start of taking the drug.
Question 3:
(1) How do you feel about ejaculation after you started taking the drug?
* No change
* Feel difference from the condition before taking the drug.
If you can explain, please specifically describe the feeling ( ).
How often do you feel the difference?
* On each time
* Approximately on 2 of 3 times
* Approximately on 1 of 2 times
* On 1 of 3 or more times
(2) How is the amount of semen at the time of ejaculation after you started taking the drug?
* No change
* The amount became decreased
* No semen at all
(3) If you are aware of the decreased amount of semen or no semen at the time of ejaculation, do you worry about it?
* I do not worry and want to continue the medication.
* I worry but want to continue the medication.
* I worry and want to discontinue the medication

In the questionnaire survey (Table 1), Question 1 is related to the adverse reactions including ejaculatory disorder after taking oral silodosin, and Question 2 investigates the presence or absence of sexual actions (sexual intercourse and masturbation) and the frequency. Question 3 is targeted at only the patients who exercised sexual actions and had the chances of ejaculation even after taking oral silodosin, more concretely, to investigate the presence or absence of ejaculatory disorder and frequency, changes and prevalence in the amount of semen at the time of ejaculation. The last question was whether or not the patients wished to discontinue oral silodosin because of the ejaculatory disorder. Each of the items including the prostate volume before silodosin administration, the international prostate symptom score (IPSS) and QOL score before and after 4 weeks from administration, were investigated in the 91 patients who responded to the questionnaire. Questionnaires were administered in this hospital and a fixed doctor has completed the questionnaires with interview form. This questionnaire survey was retrospectively performed for 91 LUTS/BPH patients who were administered silodosin for over 4 weeks, and questionnaires were given to patients at the revisit of the time all patients who already been on treatment have been contacted.

This research was approved in the ethical committee in our hospital and all patients were consented to this research. As to the statistic analysis, ANOVA, t-test and x2 test were employed, and p<0.05 was handled as significant difference.

## Results

**Figure 1:**
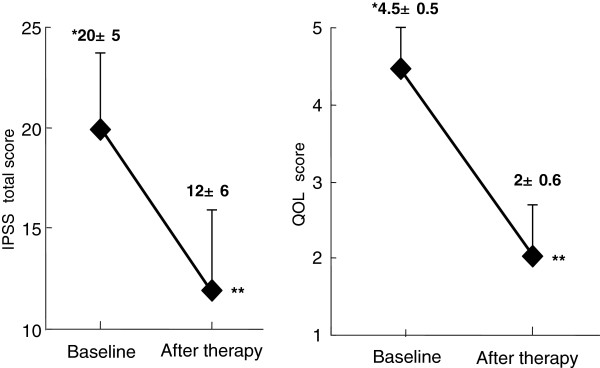
**Changes in IPSS and QOL score by the treatment with silodosin (n=91).** *Mean ± SD. **p<0.01 by Wilcoxon signed-rank test.

**Figure 2:**
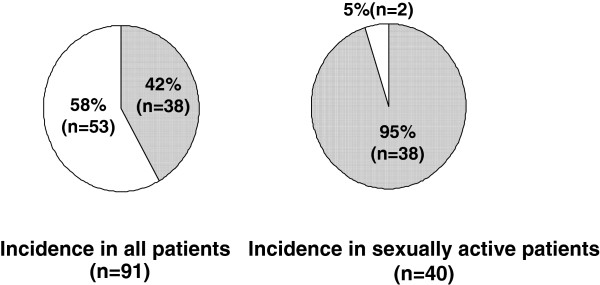
**Incidence of ejaculatory disorder in all patients and sexually active patients.** Ejaculatory disorder present (fully shaded area). ejaculatory disorder absent (non shaded area).

The questionnaire survey was conducted in the patients who had been taking oral silodosin for over 4 weeks from the start. All 91 patients completed the questionnaire survey. As to the background of 91 patients, their age was 55~ 84 years old (mean 66.9 ± 6.9), silodosin administration period was 2 ~18 months (mean 6.7 ± 2.8) and prostate volume was 31 ~94 ml (mean 39 ± 10.3). Figure 1 shows the changes in IPSS and QOL score before and after silodosin administration. Compared with the status before treatment, the IPSS and QOL score after 4 weeks of silodosin administration were significantly improved (p<0.001). Table 2 shows the results related to adverse reactions. Ejaculatory disorder occurred in 38 (42%) of the 91 patients, indicating the highest incidence among all the adverse reactions. As to the presence or absence of sexual actions (sexual intercourse, masturbation), 40 (44%) of the 91 patients carried out sexual actions even after the oral intake of silodosin. In other words, the incidence of ejaculatory disorder among those who carry out sexual actions after taking oral silodosin was as high as 95% (38/40) patients (Figure 2).

**Table 2:** Adverse reactions (n=91)

	
Loose stool/diarrhea	8 (9%)
Dizziness	2 (2%)
Headache	0
Nasal congestion	4 (4%)
Dry mouth	0
Ejaculatory disorder	38(42%)

**Figure 3:**
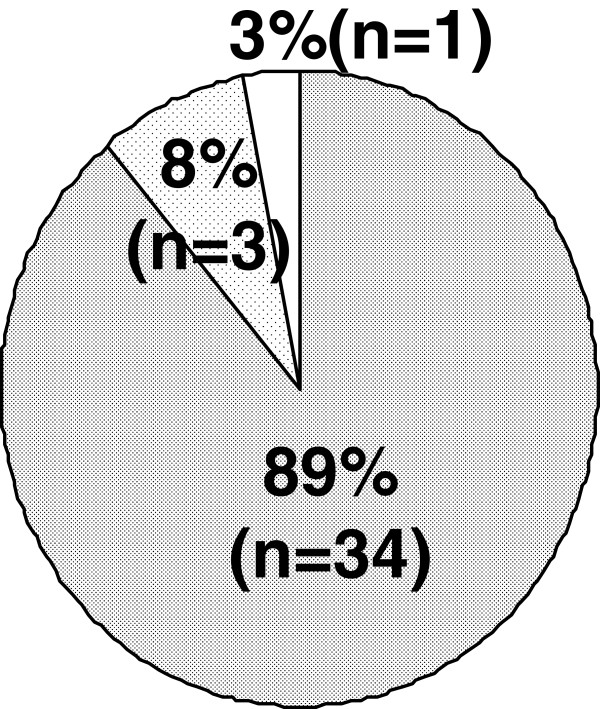
**Incidence of ejaculatory disorder in patients with ejaculatory disorder (n=38).** On each time (fully shaded area). approx. on 2 of 3 times (slightly shaded area). approx. on 1 of 2 times (non shaded area).

**Figure 4:**
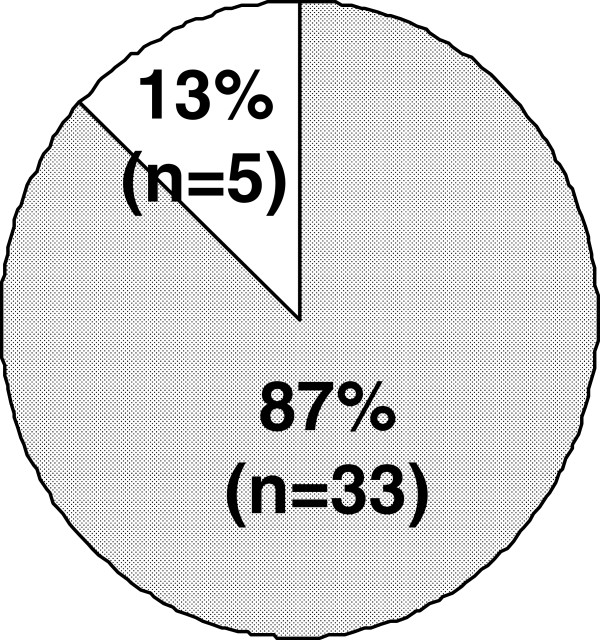
**Amount of ejaculatory semen in patients with ejaculatory disorder (n=38).** Loss of semen emission (fully shaded area). decreased semen emission (non shaded area).

**Figure 5:**
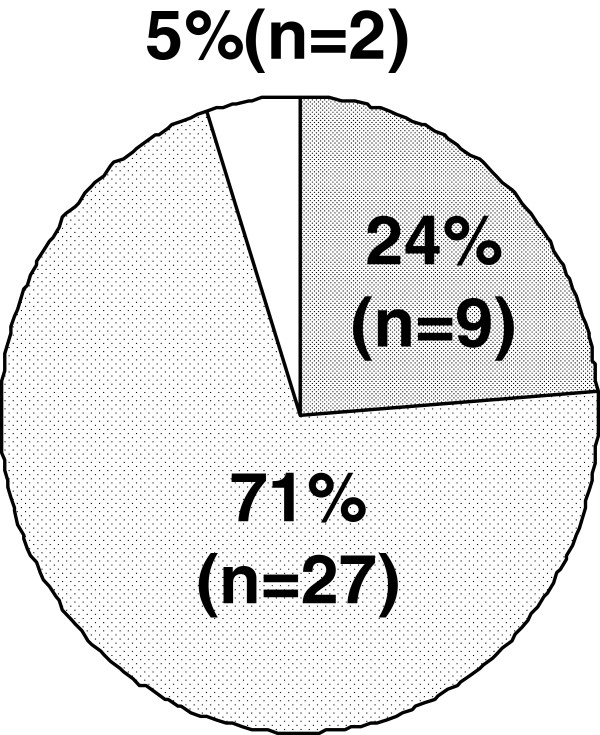
**Desire to continue or discontinue the silodosin treatment in patients with ejaculatory disorder (n=38).** I do not worry and want to continue the medication (fully shaded area). I worry but want to continue the medication (slightly shaded area). I worry and want to discontinue the medication (non shaded area).

Concerning ejaculatory disorder, the incidence among the patients who experienced ejaculatory disorder each time was 89% (Figure 3). As to the amount of semen at the time of ejaculation, the disappearance of semen was observed in 87% (Figure 4). Figure 5 shows whether or not the patients with ejaculatory disorder wished to discontinue oral silodosin. Of the 38 patients, 9 (24%) did not worry about the said disorder and wished to continue the medication, 27 (71%) worried about the said disorder but wished to continue the medication, and 2 (5%) worried about the said disorder and wished to discontinue the medication. In other words, 29 (76%) of the 38 patients worried about the ejaculatory disorder.

Oral silodosin was discontinued due to the ejaculatory disorder in 2 (5%) of the 38 patients. On the whole, the discontinuation rate of oral silodosin was 2% (2/91 patients). After discontinuation of intake of silodosin, the all patients who had ejaculatory disorder recovered their ejaculatory function. In regard to the orgasm, 20 of the 38 (53%) patients who occurred ejaculatory disorder answered the presence of orgasm.

## Discussion

According to the AUA guideline [7], the incidence of erectile dysfunction (“ED”) and hypoactive sexual desire (“HSD”) by α1 blockers such as alfuzosin, doxazosin, tamuslosin and terazosin is not much different from that by a placebo but the incidence and degree of ejaculatory disorder differ depending on the type of α1 blocker. The incidence of ejaculatory disorder is mostly reported on the basis of adverse reaction reports in the clinical trials. However, this incidence refers to the ratio among the whole patients administered α1 blockers without any consideration to the presence or absence of sexual actions (sexual intercourse, masturbation). The incidence of ejaculatory disorder among the patients who carry out sexual actions even under the treatment with oral α1 blockers should be handled as the true incidence. The incidence of ejaculatory disorder among the LUTS/BPH patients under treatment with oral silodosin is higher than that in comparison with that caused by other α1 blockers. Yokoyama et al. explored the effect of three different types of α1-blockers (tamsulosin, naftopidil, silodosin) on lower urinary tract symptoms and sexual function in patients with benign prostatic hyperplasia. They reported that all three types of α1-blockers provided an objective and subjective improvement of LUTS without any significant difference among them, but erectile function only improved in patients treated with naftopidil and a higher rate of ejaculatory disorder (24.4%) was observed in those receiving silodosin [8]. Kawabe et al. reported that ejaculatory disorder occurred in 22.3% of those treated with silodosin against 1.6% in those treated with tamuslosin [6]. The incidence of ejaculatory disorder is expected to go up further if the survey was conducted only in the patients carrying out sexual actions. The questionnaire table this time was prepared on our own and it is not a validated one. However, the result of this questionnaire indicated that the incidence of ejaculatory disorder in all the patients administered silodosin was 42% but the incidence sharply went up to 95% when only the patients carrying out sexual actions while taking oral silodosin were investigated. It is conceivable that the incidence of ejaculatory disorder may increase/decrease depending on the oral dose of α1 blocker. However, this questionnaire survey among the patients who had taken the oral silodosin at the standard dose prescribed in Japan clearly showed ejaculatory disorder was induced by silodosin at a very high rate. Furthermore, this questionnaire survey also demonstrated that the incidence of ejaculatory disorder by α1 blockers became very high if the ratio is calculated only among those who carry out sexual actions. Accordingly, it is considered necessary to include the presence or absence of sexual actions (sexual intercourse, masturbation) in the survey of the incidence of ejaculatory disorder.

Regarding the ejaculatory disorder onset mechanism by α1 blocker, the contraction disorder of seminal vesicle and spermatic duct at the time of ejaculation is assumed as a major cause [9,10]. As to the distribution of α1 receptor subtypes in the human seminal vesicle, α1A receptor, α1B receptor and α1D receptor account for 75%, 11.7% and 13.3% respectively, indicating the superiority of α1A receptor in the seminal vesicle as well as in the prostate [9]. Furthermore, it was pharmacologically acknowledged that the contraction of human spermatic duct also occurs through α1A receptor [11]. Hisasue et al. administered tamuslosin or naftopidil to adult male volunteers and quantified the ejaculation volume, fructose concentration in the semen, and sperm in urine after ejaculation. They reported that the ejaculation volume and fructose in the semen decreased, and that no sperm was observed in the urine after ejaculation by the influence of tamuslosin [9]. Hellstorm et al. reported that ejaculation completely disappeared in 17 (35.4%) of the 48 volunteers that took oral tamuslosin 0.8 mg but the urinary sperm count after ejaculation showed no change from the level before intake [10]. Based on the above, the ejection disorder due to the insufficient contraction of seminal vesicle and spermatic duct is conceivable as the mechanism of ejaculatory disorder by tamuslosin. Furthermore, Nagai et al. investigated the mechanism of ejaculatory disorder attributable to the α1-blocker silodosin, a real-time observation of ejaculation by using transrectal color Doppler ultrasonography. They reported that the mechanism of ejaculatory disorder is intricately related to retrograde ejaculation (retrograde inflow of semen fluid), insufficient contraction of the seminal vesicles, and insufficient rhythmic contraction of the muscles of the pelvic floor [12].

Furuya et al. investigated the ejaculatory disorder by assigning the BPH patients into tamuslosin group and naftopidil group, and reported that the prevalence of ejaculatory disorder was significantly higher in the tamuslosin group that demonstrated higher affinity to α1A receptor in comparison with naftopidil [13]. Furthermore, higher rate of ejaculatory disorder was observed in silodosin receiving patients compared with tamsulosin or naftopidil receiving patients [8]. Since the questionnaire survey this time disclosed that the incidence of ejaculatory disorder and the frequency of semen disappearance were high in the patients taking oral silodosin at the standard dose prescribed in Japan in this questionnaire survey, a blocker with higher affinity to α1A receptor is assumed to further increase the incidence of ejaculatory disorder as described in the above.

It is reported that the oral administration is discontinued only in a few cases because of the ejaculatory disorder by α1A blocker [6,14]. Schulman reported that the discontinuation of tamuslosin due to ejaculatory disorder occurred only in 0~0.8% on the whole, and in 0~18% even in the ejaculatory disorder cases [14]. Oral silodosin was discontinued in only 2 patients due to the ejaculatory disorder by silodosin, which accounted for 5% in the patients with ejaculatory disorder in this survey and only 2% in all the patients who took oral silodosin. Even if ejaculatory disorder occurs, discontinuation of oral silodosin is not necessarily required if the QOL of patient himself is not decreased because of this disorder. The patients who had ejaculatory disorder accept good effects of silodosin for LUTS/BPH; it may be the reason why they continue silodosin administration, even if ejaculatory disorder occurs. Homma et al. reported that the silodosin subgroup with ejaculation disorder showed larger change in total IPSS than the silodosin subgroup without ejaculation disorder [15]. Roehrborn et al. also explained that silodosin-treated patients with retrograde ejaculation experienced numerically greater improvement in IPSS and Qmax compared with silodosin-treated patients without retrograde ejaculation [16]. These results suggest that ejaculatory disorder caused by silodosin associated with very large improvements in patients with benign prostatic hyperplasia. On the other hand, 76% of the patients who gave response to the questionnaire survey in this study said that they worried about ejaculatory disorder, suggesting that, other than LUTS, the silodosin-induced ejaculatory disorder could lead to decrease the QOL of patients.

We recognized the presence of orgasm on 20 of the 38(53%) patients who occurred ejaculatory disorder receiving silodosin. The effect of silodosin on the orgasmic function of men who are administered α1 blockers for BPH is unknown. Ejaculatory disorder occurs via interference in the muscle contraction of the vas deferens and seminal vesicle, patients can still engage in sexual intercourse and experience satisfying orgasm [17]. Most retrograde ejaculation events in silodosin-treated patients (82%) were reported as “orgasm with absence of seminal emission” [16]. While Shimizu et al. reported that abnormal ejaculation that results in a disappeared or decreased of semen and reduced contraction of the bulbocavernosus/pelvic floor muscles as a result of silodosin administration may decrease the subjective pleasure of orgasm [18], confirmed Nagai et al. observed insufficient rhythmic contraction of pelvic floor muscles using transrectal color Doppler ultrasonography in dry ejaculation caused by silodosin administration [12]. Semen passing through the urethra and sufficient rhythmic contraction of pelvic floor muscles may contribute to orgasmic function. However ejaculatory disorder may be tolerable adverse reaction, if the orgasm is unchanged and LUTS is improved.

Though not all the prostate hyperplasia patients are expected to carry out sexual actions, it is still considered necessary to provide sufficient information on the ejaculatory disorder as an adverse reaction before starting the treatment with silodosin. However, further caution is required for the administration of α1 blocker to young patients. In comparison with the elderly, there is no doubt that the ejaculatory disorder in the young patients causes serious decline in QOL.

The present analyses and conclusion have limitations because of having no controls or placebo group. To confirm these results, we need a large prospective clinical trial having placebo-control group. Furthermore, it is necessary to elucidate the prevalence and mechanism of ejaculatory disorder and orgasmic function by α1 blockers furthermore in the future.

## Conclusions

It was demonstrated that the administration of silodosin induced ejaculatory disorder at a high incidence among the patients who exercised sexual actions. Though not all the prostate hyperplasia patients are expected to carry out sexual actions, it is still considered necessary to provide sufficient information on the ejaculatory disorder as an adverse reaction before starting the treatment with silodosin.

## Competing interests

The authors declare that they have no competing interests.

## Authors’ contributions

KS and TM drafted the report, cared for the patients and approved the final version of the manuscript.

## Pre-publication history

The pre-publication history for this paper can be accessed here:

http://www.biomedcentral.com/1471-2490/12/29/prepub

## References

[B1] Kojima Y Sasaki S Hayashi Y Tsujimoto G Kohri K Subtypes of alpha1-adrenoceptors in BPH: future prospects for personalized medicine Nat Clin Pract Urol 2009 6 44 53 10.1038/ncpuro1276 19132005

[B2] Schwinn DA Roehrborn CG α 1-adrenoceptor subtypes and lower urinary tract symptoms Int J Urol 2008 15 193 99 10.1111/j.1442-2042.2007.01956.x 18304211 PMC2329815

[B3] Komiya A Suzuki H Awa Y Egoshi K Ohnishi T Nakatsu H Ohki T Mikami K Sato N Araki K Ota S Naya Y Ichiwaka T Clinical effect of naftopidil on the quality of life of patients with lower urinary tract symptoms suggestive of benign prostatic hyperplasia: a prospective study Int J Urol 2010 17 555 62 10.1111/j.1442-2042.2010.02518.x 20370847

[B4] Nishino Y Masue T Miwa K Takahashi Y Ishihara S Deguchi T Comparison of two alpha1-adrenoceptor antagonists, naftopidil and tamsulosin hydrochloride, in the treatment of lower urinary tract symptoms with benign prostatic hyperplasia: a randomized crossover study BJU Int 2006 97 747 51 10.1111/j.1464-410X.2006.06030.x 16536766

[B5] Yoshida M Kudoh J Homma Y Kawabe K New clinical evidence of silodosin, an α (1A) selective adrenoceptor antagonist, in the treatment for lower urinary tract symptoms Int J Urol 2012 19 306 16 10.1111/j.1442-2042.2011.02957.x 22251148

[B6] Kawabe K Yoshida M Homma Y Silodosin, a new α1A-adrenoceptor-selective antagonist for treating benign prostatic hyperplasia: results of a phase III randomized, placebo-controlled, double-blind study in Japanese men BJU Int 2006 98 1019 1024 10.1111/j.1464-410X.2006.06448.x 16945121

[B7] Practice AUA Guidelines Committee: AUA guideline on management of benign prostatic hyperplasia. Chapter 1. Diagnosis and treatment recommendations J Urol 2003 170 530 547 12853821 10.1097/01.ju.0000078083.38675.79

[B8] Yokoyama T Hara R Fukumoto K Jo Y Miyaji Y Nagai A Sone A Effects of three types of alpha-1 adrenoceptor blocker on lower urinary tract symptoms and sexual function in males with benign prostatic hyperplasia Int J Urol 2011 18 225 30 10.1111/j.1442-2042.2010.02708.x 21272091

[B9] Hisasue S Furuya R Itoh N Kobayashi K Furuya S Tsukamoto T Ejaculatory disorder caused by alph-1 adrenoceptor antagonists is not retrograde ejaculation but loss of seminal emission Int J Urol 2006 13 1311 16 10.1111/j.1442-2042.2006.01535.x 17010010

[B10] Hellstorm WJG Sikka SC Effects of acute treatment with tamsulosin versus alfuzosin on ejaculatory function in normal volunteers J Urol 2006 176 1529 33 10.1016/j.juro.2006.06.004 16952675

[B11] Moriyama N Nasu K Takeuchi T Akiyama K Murata S Nishimatsu H Yano J Tsujimoto G Kawabe K Quantification and distribution of α1-adrenoceptor subtype mRNAs in human vas deferens: comparison with those of epididymal and pelvic portions Br J Pharmacol 1997 122 1009 14 10.1038/sj.bjp.0701485 9401762 PMC1565044

[B12] Nagai A Hara R Yokoyama T Jo Y Miyaji Y Ejaculatory dysfunction caused by the new alpha1-blocker silodosin: A preliminary study to analyze human ejaculation using color Doppler ultrasonography Int J Urol 2008 15 915 18 10.1111/j.1442-2042.2008.02136.x 18721206

[B13] Furuya R Hisasue S Ogura H Furuya S Masumori N Itoh N Tsukamoto T Ejaculatory disorder by alpha-1 adrenoceptor antagonist in patients with benign prostatic hyperplasia comparison between naftopidil and tamsulosin Hinyokika Kiyo 2005 51 763 766 16363711

[B14] Schulman CC Lower urinary tract symptoms/ benign prostatic hyperplasia: minimizing morbidity caused by treatment Urology 2003 62 24 33 12957197 10.1016/s0090-4295(03)00471-0

[B15] Homma Y Kawabe K Takeda M Yoshida M Ejaculation disorder is associated with increased efficacy of silodosin for benign prostatic hyperplasia Urology 2010 76 1446 50 10.1016/j.urology.2010.03.015 20472263

[B16] Roehrborn CG Kaplan SA Lepor H Volinn W Symptomatic and urodynamic responses in patients with reduced or no seminal emission during silodosin treatment for LUTS and BPH Prostate Cancer Prostatic Dis 2011 14 143 8 10.1038/pcan.2010.46 21135869 PMC3094762

[B17] Sanbe A Tanaka Y Fujiwara Y Tsumura H Yamauchi J Cotecchia S Koike K Tsujimoto G Tanoue A Alpha1-adrenoceptors are required for normal males sexual function Br J Pharmacol 2007 152 332 40 17603545 10.1038/sj.bjp.0707366 PMC2042949

[B18] Shimizu F Taguri M Matsuyama Y Sase K Fujime M Impact of dry ejaculation caused by highly selective alpha1A-blocker: randomized, double-blind, placebo-controlled crossover pilot study in healthy volunteer men J Sex Med 2010 7 1277 83 10.1111/j.1743-6109.2009.01663.x 20102447

